# MicroRNA networks in prolactinoma tumorigenesis: a scoping review

**DOI:** 10.1186/s12935-024-03529-5

**Published:** 2024-12-19

**Authors:** Sevil Ghaffarzadeh Rad, Fatemeh Nejadi Orang, Mahdi Abdoli Shadbad

**Affiliations:** 1 Research Center for Evidence-based Medicine, Iranian EBM Centre: A JBI Centre of Excellence, Faculty of Medicine, Tabriz University of Medical Sciences, Tabriz, Iran; 2 Immunology Research Center, Tabriz University of Medical Sciences, Tabriz, Iran; 3 Department of Immunology, Tabriz University of Medical Sciences, Tabriz, Iran

**Keywords:** CeRNA, CircRNA, LncRNA, MicroRNAs, Prolactinoma

## Abstract

**Background:**

Prolactinoma is the leading type of pituitary adenoma. Aside from the mass-like effect of prolactinoma, its hormonal effect is the main pathological cause of endocrine dysregulation and infertility. The dopamine agonist administration and surgical resection are the current mainstream anti-neoplastic treatments for affected patients; however, tumor fibrosis, tumor invasion, dopamine agonist resistance, and gain prolactinomas are clinical challenges for treating affected patients. Therefore, there is a need to develop novel treatments for these patients. Although growing evidence has highlighted the significance of dysregulated microRNA (miRNA) expression in various malignancies, no study has systematically investigated the significance of miRNA networks and their therapeutic potential in prolactinoma. For this aim, the current scoping review was performed according to the systematic reviews and meta-analyses extension for scoping reviews (PRISMA-ScR) guideline.

**Main body:**

The systematic study on PubMed, Web of Science, Scopus, and Embase databases has shown that miR-200c, miR-217, miR-93a, miR-93, miR-1299, and miR-9 are the oncogenic miRNAs and miR-137, miR-145-5p, miR-197-3p, miR-29a-3p, miR-489, miR-199a-5p, miR-124, miR-212, miR-129-5p, miR-130a-3p, miR-326, miR-432, miR-548c-3p, miR-570, miR-15, miR-16, miR-26a, miR-196a2, and let-7a are tumor-suppressive miRNAs in prolactinoma tumorigenesis.

**Conclusion:**

In summary, inhibiting the oncogenic miRNAs and ectopic expression of tumor-suppressive miRNAs can decrease prolactin secretion, reduce tumor invasion and migration, enhance dopamine agonist efficacy, and inhibit prolactinoma development. These findings can serve as a blueprint for future translational studies investigating miR-based therapeutics for prolactinoma.

**Graphical Abstract:**

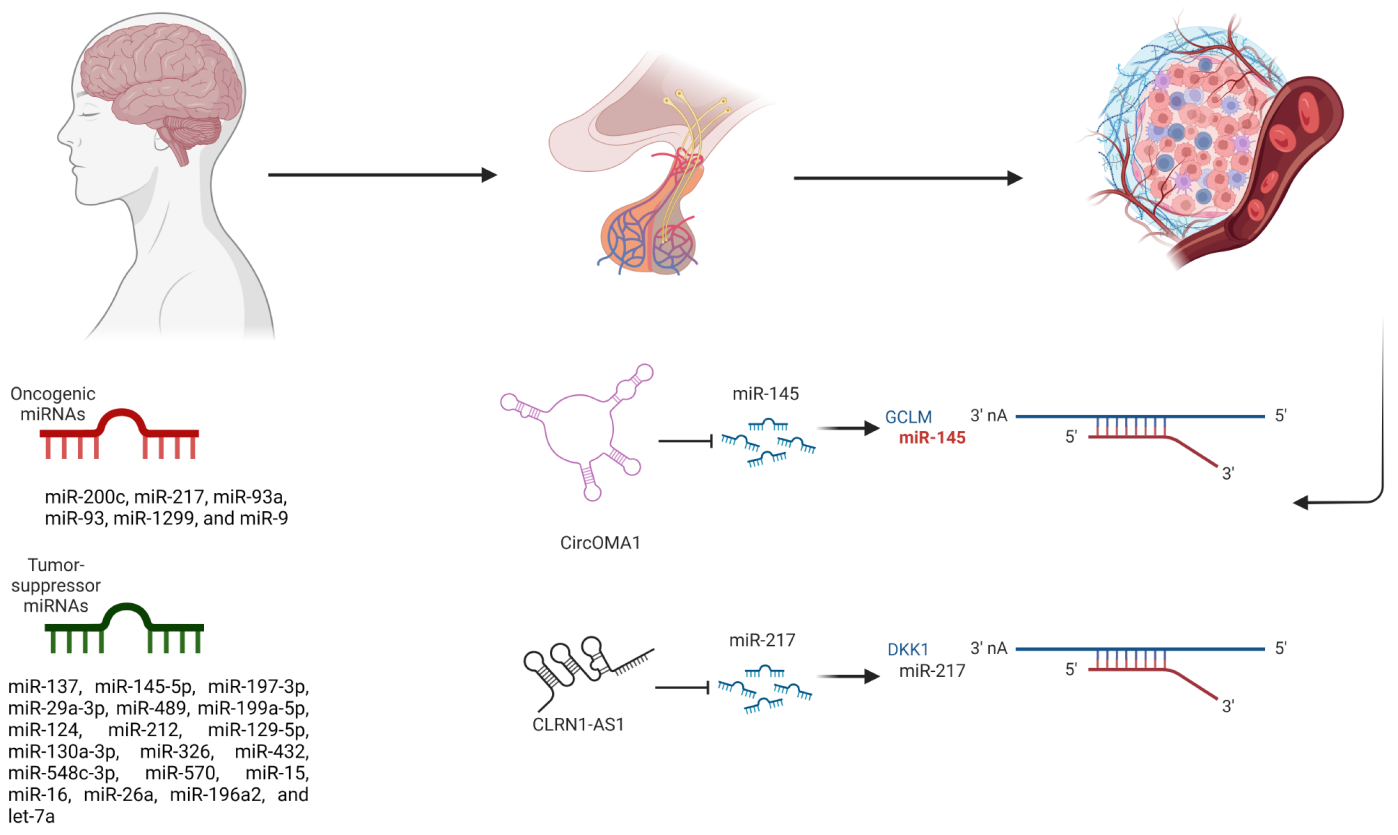

## Introduction

Pituitary adenomas are non-malignant neoplasms; however, as the third most common brain tumor, they carry profound health burden [1]. Based on recent data from the Central Brain Tumor Registry of the United States, the incidence of pituitary lesions was 4.07 cases per 100,000 per year between 2012 and 2016. Also, the prevalence of clinically relevant pituitary adenomas is 89.1 per 100,000. As the leading type of pituitary adenoma, prolactinoma is responsible for approximately 50% of all pituitary adenomas [2]. The burden of prolactinoma is well-established in the fields of endocrinology, gynecology, neurology, neurosurgery, and reproductive medicine given its hormonal and mass effects [3]; therefore, prolactinoma is considered a significant primary brain tumor that requires medical intervention.

Based on the recently published consensus statement by Petersenn et al., dopamine agonists, particularly cabergoline, are highly effective in lowering serum prolactin levels, reducing tumor size, and improving the clinical manifestation of patients with prolactin-secreting pituitary adenomas. However, resistance to and intolerance of dopamine agonists are indications for surgery in macroprolactinoma patients [4]. According to a retrospective study conducted by Vermeulen et al., dopamine agonist resistance was found to be 15.9% among patients with prolactinomas [5]. Besides, tumor resection is not always straightforward; tumor fibrosis makes complete resection of macroprolactinomas with fibrosis challenging [6]. Also, surgery for giant invasive prolactinomas aims to alleviate the mass effect of the tumor rather than cure it [7]. Therefore, a deeper understanding of the biology of prolactinoma tumorigenesis can offer novel therapeutic options for these lesions.

Although protein-coding genes constitute an integral part of the genome, a considerable part of the human genome is made up of non-protein-coding genes [8, 9]. These non-coding genes extensively regulate the signaling pathways [10]. It has been reported that the extent of protein-coding sequences does not increase appropriately across vertebrates; however, the non-coding genes extent increases with organism complexity [11]. As a well-studied non-coding RNA, microRNA (miRNA) dysregulation is considerably implicated in oncogenesis [12, 13]; these small non-coding RNAs consist of approximately 22 nucleotides that post-transcriptionally regulate their target messenger RNAs (mRNAs) both directly and indirectly [14]. The RNA polymerase II/III-mediated transcription of miRNA genes produces primary miRNAs [15]. The Drosha and DiGeorge critical region-8 (DGCR8) complex-mediated processing of primary miRNAs produces precursor miRNAs in the nucleus [16]. Afterward, exportin 5 transports precursor miRNAs to the cytoplasm, and the processing mediated by Dicer produces mature miRNAs [17]. The loading of mature miRNAs to miRNA-induced silencing complex paves the way for the target mRNA degradation and expression inhibition through a complementary binding manner [18, 19]. The 3’ untranslated regions of target mRNA are the main route through which miRNAs exert their regulatory function of miRNAs on their target mRNA genes [20]. Given the fact that a single miRNA can have a large number of direct target genes, their dysregulation massively affects cellular signaling pathways [21]. The downregulation of tumor-suppressive miRNAs disinhibits the expression of oncogenes, while the upregulation of oncogenic miRNAs decreases the expression of tumor-suppressive genes, paving the way for tumor development and progression [22]. Aside from this, Salmena et al. have shed light on the interplay between miRNAs with other non-coding RNAs [23]. The “language” between these non-coding RNAs is considered microRNA response elements (MREs); MREs are present in circular RNA (circRNAs), long non-coding RNA (lncRNA), and mRNAs and this constitutes a dynamic competition for binding the pool of specific miRNAs [24]. This concept gives rise to the newly identified regulatory networks, i.e., the competing endogenous RNA (ceRNA) networks, that govern cellular behavior [25]. Growing research has identified the significance of circRNA/miR/mRNA and lncRNA/miR/mRNA axes in different human malignancies development and progression [26–28]. Although miRNAs and ceRNA networks can have a significant impact on cell behavior and their dysregulation has been implicated in various aspects of tumorigenesis, no study has thoroughly investigated microRNA networks in prolactinoma tumorigenesis.

This scoping review aimed to systematically study the existing evidence on the significance of miRNAs in prolactinoma tumorigenesis and treatment. For this aim, we systematically reviewed the studies that investigated the effect of miRNA on various aspects of prolactinoma development via experimental assays. We also shed light on the discovered lncRNA- and circRNA-mediated ceRNA networks in prolactinoma tumorigenesis. The identified tumor-suppressive miRNAs and oncogenic miRNAs can pave the way to address the current challenges in treating dopamine agonist-resistant and invasive prolactinomas in affected patients.

## Method

### Scoping review protocol

The current scoping review adheres to the systematic reviews and meta-analyses extension for scoping reviews (PRISMA-ScR) guideline [29]. Formulating the research question, identifying pertinent publications, selecting studies, charting the data, and summarizing and reporting the findings comprise the current scoping review’s five stages.

### Research question

The present scoping review aimed to investigate the significance of miRNAs in the development and treatment of prolactinoma. Also, we studied the identified lncRNA- and circRNA-mediated ceRNA networks in prolactinoma tumorigenesis.

### Finding relevant publication

A systematic search was conducted in the Web of Science, Scopus, PubMed, and Embase to identify pertinent studies published prior to March 28, 2024; language, country, and time restrictions were not imposed on the systematic searches. Prolactinoma and miRNA, along with their different versions, Emtree, and Medical Subject Headings (MeSH) terms, were used to construct the syntax for the systematic search.

### Study selection

After extracting publications from the aforementioned databases and removing duplicate records, the papers went through a two-phase review process. The initial stage involved evaluating the titles and abstracts of the retrieved studies. In the second phase, the full texts of the remaining papers underwent a comprehensive evaluation. The subsequent items served as inclusion criteria are as follows. First, the study must be an original article published in English. Second, the included study needed to experimentally investigate the importance of miRNA in prolactinoma tumorigenesis. The studies also must include functional experiments to study the effects of the studied miRNA on various aspects of tumorigenesis such as proliferation, migration, invasion, drug resistance, etc.

### Data charting

The studied miRNAs, the prolactinoma cell line, and the effect of the studied miRNAs on prolactinoma tumorigenesis, along with the ceRNA networks, if present, were extracted from the included studies.

### Summarizing and reporting the findings

The current scoping review summarized the available evidence on the significance of the identified miRNAs in prolactinoma tumorigenesis and treatment.

## Results

### Systematic search results

**Fig. 1:**
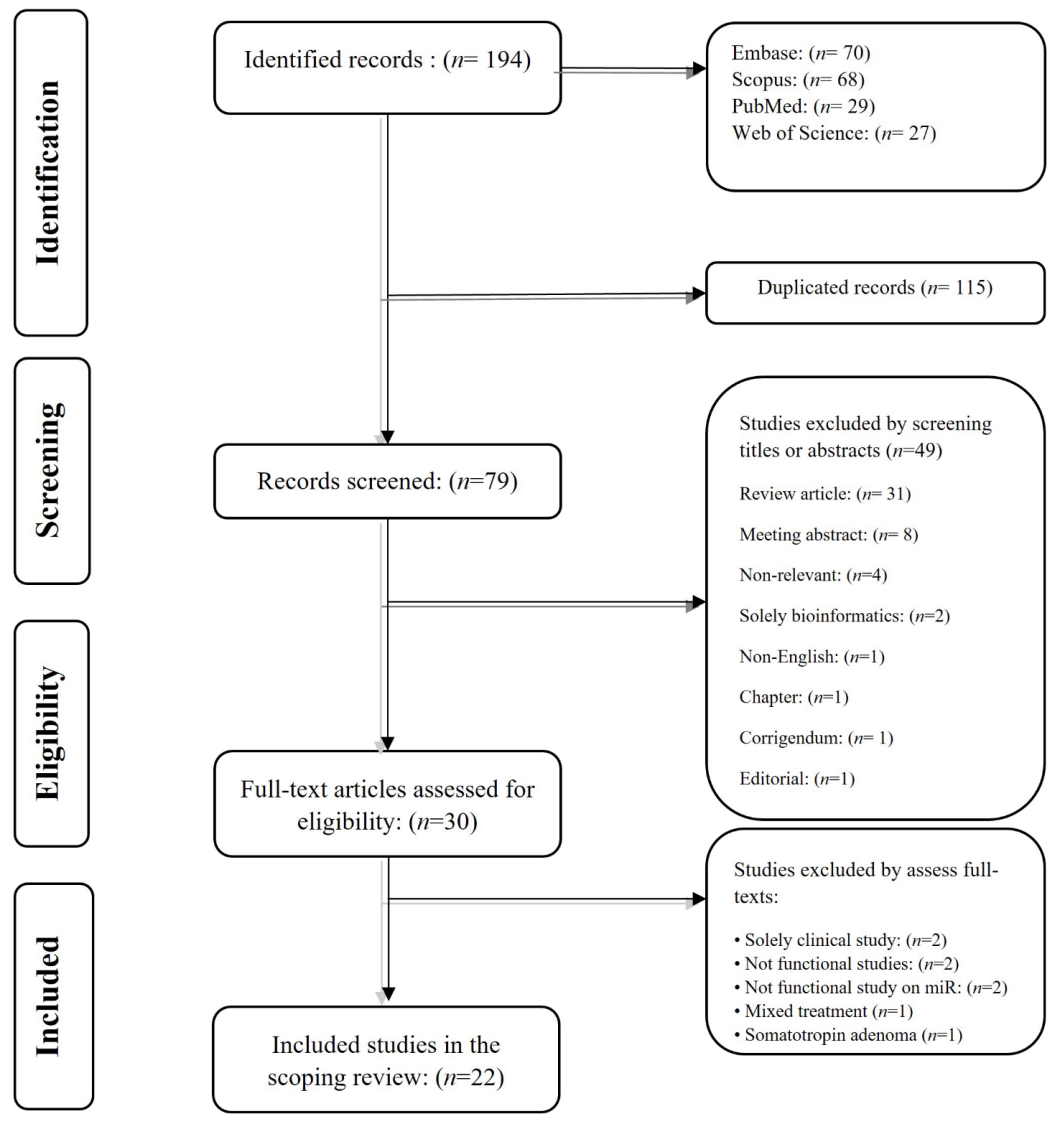
The flow chart of the study

The systematic search on the PubMed, Web of Science, Scopus, and Embase databases retrieved 194 publications. Following the removal of duplicate studies, the remaining records were screened based on their titles and abstracts. Review articles, meeting abstracts, book chapters, and editorials were among the excluded studies. Afterward, the full text of the remaining studies was thoroughly reviewed. Ultimately, 22 original studies that met the above-mentioned criteria were included in this scoping review. The flowchart of the inclusion and exclusion of studies is shown in Fig. 1.

### The description of the included studies

The included papers were published between 2012 and 2023. MMQ and GH3 are the commonly used prolactinoma cell lines used in the studies. It has been found that miR-200c, miR-217, miR-93a, miR-93, miR-1299, and miR-9 are the identified oncogenic miRNAs in prolactinoma. With being validated in independent studies, miR-93 is the most studied oncogenic miRNA in prolactioma. Regarding ceRNA networks, CLRN1 antisense RNA 1 (CLRN1-AS1)/miR-217/ Dickkopf-related protein 1 (DKK1) and H19/miR-93a/autophagy related 7 (ATG7) are the identified tumor suppressive ceRNA that suppresses miR-217 and miR-93a, respectively (Table 1).

miR-137, miR-145-5p, miR-197-3p, miR-29a-3p, miR-489, miR-199a-5p, miR-124, miR-212, miR-129-5p, miR-130a-3p, miR-326, miR-432, miR-548c-3p, miR-570, miR-15, miR-16, miR-26a, miR-196a2, and let-7a are the identified tumor-suppressive miRNAs in prolactinomas. miR-137 and miR-145 are the tumor-suppressive miRNAs that have been validated in independent studies. The circOMA1/miR-145-5p/glutamate-cysteine ligase modifier subunit (GCLM) axis is the only experimentally validated ceRNA network that has oncogenic effects by suppressing miR-145-5p expression in prolactinoma (Table 1).

**Table 1:** microRNAs in prolactinoma tumorigenesis

Category	MicroRNA	Cell line	ceRNA network	Reference
Oncogenic microRNAs	miR-200c	MMQ and GH3	Not studied	[30]
	miR-217	PPA	CLRN1-AS1/miR-217/DKK1	[31]
	miR-93a	GH3	H19/miR-93a/ATG7	[32]
	miR-93	MMQ and GH3	Not studied	[33]
	miR-93	MMQ	Not studied	[34]
	miR-93-5p	MMQ	Not studied	[35]
	miR-1299	MMQ	Not studied	[36]
	miR-9	MMQ	Not studied	[37]
Tumor-suppressive microRNAs	miR-137	MMQ	Not studied	[38]
	miR-137	MMQ and GH3	Not studied	[39]
	miR-145-5p	MMQ	CircOMA1/miR-145-5p/GCLM	[40]
	miR-145-5p	MMQ	Not studied	[41]
	miR-29a‑3p	MMQ and GH3	Not studied	[42]
	miR-197-3p	GH3	Not studied	[43]
	miR-489	MMQ and GH3	Not studied	[44]
	miR-199a-5p	MMQ	Not studied	[45]
	miR-124	MMQ and GH3	Not studied	[46]
	miR-212	GH3	Not studied	[47]
	miR-129-5p	MMQ	Not studied	[48]
	miR-130a-3p	GH3	Not studied	[49]
	miR-326, miR-432, miR-548c-3p, and miR-570	GH3	Not studied	[50]
	miR-15, miR-16, miR-26a, miR-196a2, and let-7a	GH3	Not studied	[51]

*Abbreviation* miR: microRNA

## Discussion

Although dopamine agonists, especially cabergoline, have well-established clinical benefits for the majority of patients [52], tumor invasion, drug resistance, tumor fibrosis, and giant prolactinomas are considerable obstacles to treating patients with these therapeutic approaches [53]. Therefore, there is a need to better understand the biology of prolactinoma to develop novel therapeutic options. The following discusses the currently available evidence on the significance and therapeutic potentiality of miRNAs in the development and treatment of prolactinomas.

As an upregulated lncRNA in dopamine agonist-sensitive prolactinomas, H19 overexpression enhances the sensitivity of prolactinoma cells to cabergoline and bromocriptine via the H19/miR-93a/ATG7. Also, combined cabergoline treatment with H19 decreases tumor growth in vivo; however, miR-93a mimics significantly increase prolactinoma growth in vivo [32]. In line with this, it has been reported that H19 is a downregulated lncRNA in prolactinoma tissues; H19 ectopic expression decreases cell proliferation in vitro and tumor growth in vivo; the increased expression of H19 has been superior to the anti-tumoral effect of cabergoline. The anti-tumoral effect of H19 was independent of miR-675 expression and was through the H19-mTOR-4E-BP1 axis [54]. Through the CLRN1-AS1/miR-217/DKK1 axis, the ectopic expression of CLRN1-AS1 suppresses the cell proliferation and clonogenicity, increases caspase-3 activity, decreases tumor growth in vivo, and inhibits the Wnt/β-catenin signaling pathway. Also, it has been shown that FOXP1 is the transcriptional suppressor for CLRN1-AS1 [31]. It has been found that miR-93 mimics target ATG7 and increase the resistance to cabergoline; however, miR-93 inhibitors decrease tumor growth and increase the efficacy of cabergoline against prolactinoma in vivo [33]. Consistent with this, it has been shown that miR-93 suppresses the expression of p21 and increases the resistance of prolactinoma cells to bromocriptine and cabergoline [34]. Regarding prolactin expression, miR-93-5p mimics increase prolactin expression and attenuate the effect of bromocriptine on prolactin downregulation in prolactinoma cells [35]. In addition, miR-1299 increases the synthesis and secretion of prolactin in bromocriptine-resistant prolactinoma cells [36]. Also, miR-9 increases the prolactin expression in the tumoral cells [37]. However, it has been reported that miR-130a-3p decreases the expression of prolactin in prolactinoma cells [49].

The mitogen-activated protein kinase (MAPK)/extracellular signal-regulated kinase (ERK), and phosphoinositide 3-kinase (PI3K)/AKT signaling pathways are among the well-studied oncogenic pathways in various malignancies [55–57]. It has been reported that these two pathways are activated in pituitary adenomas and prolactinomas; therefore, inhabiting these pathways can provide therapeutic opportunities for prolactinoma treatment [58, 59]. In this regard, miR-29a-3p targets insulin-like growth factor 1 (IGF1) (as a factor for stimulating insulin-like growth factor 1 receptor (IGF1R) that is linked with rat sarcoma (RAS)-MAPK and PI3K-AKT signaling pathways), downregulates prolactin expression, increases apoptosis, suppresses clonogenicity, and inhibits the proliferation of prolactinoma cells [42]. It has been shown that miR-212 targets cellular mesenchymal-epithelial transition factor (c-Met) (a receptor tyrosine kinase that can activate the MAPK pathway), and its ectopic expression decreases the invasion and proliferation of prolactinoma cells [47]. Also, miR-197-3p suppresses ERK (a signaling factor of the MAPK pathway) in prolactinoma cells, decreases proliferation and clonogenicity, and enhances the apoptosis of tumoral cells [43]. Wang et al. have reported that the transfection of miR-200c decreases the caspase-3 activity and inhibits apoptosis rates in prolactinoma cells partly by downregulating phosphatase and TENsin homolog deleted on chromosome 10 (PTEN), a negative regulator of the PI3K/AKT pathway [30]. Xu et al. have shown that miR-137 targets the AKT2 and counteracts the proliferative properties of AKT2, a singling factor of the PI3K/AKT pathway, in prolactinoma cells [38]. In line with this, it has been reported that miR-137 is downregulated in prolactinomas compared to normal human pituitary tissues; also, miR-137 is downregulated in invasive prolactinomas compared to non-invasive ones. High expression of miR-137 is associated with improved recurrence-free survival of prolactinoma patients. In vitro assays have indicated that ectopic expression of miR-137 decreases the invasion and proliferation of prolactinoma cells and enhances their apoptosis rate via targeting melanocyte inducing transcription factor (MITF) [39]. In addition, it has been shown that miR-326, miR-432, miR-548c-3p, miR-570 ectopic expression arrests the cell cycle and decreases the proliferation of prolactinoma cells. Besides, miR-326 overexpression decreases the colony numbers of prolactinoma cells [50]. Also, it has been reported that miR-15, miR-16, miR-26a, miR-196a2, and let-7a mimics inhibit the proliferation and clonogenicity of prolactinoma cells [51].

It has been found that circOMA1 is upregulated in drug resistance prolactinoma tissues compared to sensitive ones, and its high expression is associated with increased prolactin levels. Through the circOMA1/miR-145-5p ceRNA network, circOM1 ectopic expression increases the proliferation and clonogenicity of prolactinoma cells and attenuates cabergoline-induced ferroptosis both in vivo and in vitro [40]. As a downregulated miRNA in prolactinoma tissues, miR-145-5p is also downregulated in bromocriptine-resistant prolactinoma tissues and cell lines compared to sensitive ones; miR-145-5p transfection sensitizes prolactinoma cells to bromocriptine, increases their apoptosis rate, and decreases tumor growth in vivo [41]. miR-489 mimics directly bind to PAK3 and inhibit the migration, invasion, proliferation, and clonogenicity of prolactinoma cells in vitro [44]. It has been reported that miR-129-5p suppresses the proliferation and increases the apoptosis rate of prolactinoma cells [48]. As a downregulated miRNA in prolactinomas, miR-199a-5p increases the apoptosis rate and decreases the proliferation of the prolactinoma cells [45]. Also, miR-124 ectopic expression decreases the migration, proliferation, and invasion of prolactinoma cells in vitro via targeting PHD finger protein 19 (PHF19) [46].

The present scoping review has several strengths. First, it is the first study to thoroughly investigate the extent and scope of miRNA networks in prolactinoma tumorigenesis. Second, this study also discussed the emerging role of circRNA- and lncRNA-associated ceRNA networks in prolactinoma tumorigenesis. Third, the findings of this study can be used for developing novel therapeutic approaches for patients. However, the current scoping review also suffers from several limitations as well. For instance, we did not include papers that were not published in English. Second, there might be studies that were not indexed in the major multidisciplinary /life science databases, i.e., Scopus, Embase, PubMed, and Web of Science.

## Recommendations and future perspectives

Although dopamine agonists and tumor resection have been associated with improved clinical outcomes, drug resistance, tumor invasion, and giant prolactinomas are still clinical challenges for affected patients [53]. The remarkable advances in our understanding of the regulatory non-coding RNA networks and their considerable roles in cellular behavior have opened an ever-growing research field [60]. In this regard, miRNAs are considered the pivotal elements of the ceRNAs and post-transcriptional gene regulation [61]. Although miRNA-based therapies are still in their infancy, growing studies have identified their oncogenic or tumor-suppressive properties in various human tumors and their significance in tumorigenesis [62]. As discussed above, the antagonizing oncogenic miRNAs and delivery of tumor-suppressive miRNAs into prolactinoma cells have shown remarkable anti-tumoral effects in suppressing cancer hallmarks and improving the response rates of anti-cancer therapies. Although specific and effective miRNA delivery to tumoral cells is still under investigation [63], combining these results with the miRNA delivery systems can open a new chapter for patients with aggressive prolactinoma.

## Conclusion

The current literature has indicated that miR-137, miR-145-5p, miR-197-3p, miR-29a-3p, miR-489, miR-199a-5p, miR-124, miR-212, miR-129-5p, miR-130a-3p, miR-326, miR-432, miR-548c-3p, miR-570, miR-15, miR-16, miR-26a, miR-196a2, and let-7a are tumor-suppressive miRNAs. Also, the current evidence has been found that miR-200c, miR-217, miR-93a, miR-93, miR-1299, and miR-9 are the oncogenic miRNAs in prolactinoma. Ectopic expression of these tumor-suppressive miRNAs and inhibiting these oncogenic miRNAs have therapeutic potential for treating and increasing the efficacy of dopamine agonists for prolactinoma.

## Data Availability

No datasets were generated or analysed during the current study.

## Abbreviations

PRISMA-ScR: systematic reviews and meta-analyses extension for scoping reviews
miRNA: microRNA
circRNA: circular RNA
lncRNA: long non-coding RNA
MRE: MicroRNA response element
MAPK: Mitogen-activated protein kinase
ATG7: Autophagy related 7
CLRN1-AS1: CLRN1 antisense RNA 1
DKK1: Dickkopf-related protein 1
ERK: Extracellular signal-regulated kinase
PI3K: Phosphoinositide 3-kinase
IGF1R: Insulin-like growth factor 1 receptor
IGF1: Insulin-like growth factor 1
c-Met: Mesenchymal-epithelial transition factor
PTEN: Phosphatase and TENsin homolog deleted on chromosome 10
MITF: Melanocyte inducing transcription factor
PAK3: p21-activated kinase 3
PHF19: PHD finger protein 19
DGCR8: DiGeorge critical region-8
mTOR: mammalian target of rapamycin
mRNA: messenger RNA
GCLM: Glutamate-cysteine ligase modifier subunit
RAS: Rat sarcoma
